# Renoprotective activity of anethole- rich fraction from aromatic herbs on junk food induced diabetic nephropathy in rats

**DOI:** 10.1007/s40200-022-01101-4

**Published:** 2022-08-12

**Authors:** Chitra Vellapandian, Rini R, Dinesh Sangarran Ramachandram

**Affiliations:** 1 Department Of Pharmacology, SRM College Of Pharmacy, SRM Institute of Science and Technology, 603203 Kattankulathur, Chengalpet, India; 2 Monash School of Pharmacy, Monash University, Jalan Lagoon Selatan, 47500 Bandar Sunway, Selangor Darul Ehsan, Monash, Malaysia

**Keywords:** Anethole, Diabetic nephropathy, Glycometabolism, Junk food, Oxidative stress

## Abstract

**Purpose:**

This study was carried out to study the effect of anethole rich fraction on the Diabetic Nephropathy (DN) rats, and explore the mechanisms.

**Methods:**

Male wistar rats were grouped into 4 (n = 6 per group): Control, junk food fed DN induced rats, low dose and high dose groups. DN was induced by oral junk food feeding. They were monitored for blood glucose levels and urine protein content at regular intervals. At the end of the study, the biological and hematological parameters were measured. Pancreatic and kidney viscera were taken to observe histopathological changes.

**Results:**

Both the doses of anethole rich fraction could drastically lower blood glucose levels, Low Density Lipoprotein (LDL), Glycated Serum Protein (GSP), Total Cholesterol (TC), Triglycerides (TG) (P < 0.01), Malondialdehyde (MDA) level (P < 0.01 or P < 0.05), increases insulin level (P < 0.01), High Density Lipoproteins (HDL), Glutathione Reductase (GSH) and Superoxide Dismutase (SOD) (P < 0.01 or P < 0.05 or P > 0.05). Both doses of anethole rich fraction also improved the pathological changes of kidney and pancreatic tissues in DN rats (P < 0.01 or P < 0.05 or P > 0.05).

**Conclusions:**

Hence it can be hypothesized that the high dose of anethole rich fraction (100 mg/kg) could reach the intervention effect and could ameliorate renal damage in DN rats by improving the renal functions, oxidative stress levels, glycometabolism and pathological changes of pancreas and kidney.

## Introduction

Diabetic nephropathy (DN) (diabetic kidney disease) is the widespread cause of illness and mortality among end-stage kidney disease among patients with diabetes. Major complications due to diabetic are now a worldwide health issue and the major reason of increasing rate of fatality. Increased blood glucose levels due to diabetes can lead to damaging the kidney parts which filters in the blood. Thus the damaged filter part of the kidneys becomes ‘leaky’ and allows the protein along with the urine [1]. Loss of protein due to excessive damage of glomeruli results in a lower albumin levels in blood serum causing swelling all over the body (edema), resulting in the nephrotic syndrome [2, 3]. Diabetic nephropathy is considered to the most common and one of the most serious issues in both types of diabetes mellitus and a most common cause of end stage renal disease (ESRD) across the globe [4]. Advancement of DN is characterized by structural abnormalities of renal parts such as hypertrophy of glomerular membrane, thickening of tubular glomerular basement membrane, expansion of mesangial matrix leading to the presence of albumin in excreted urine, increased creatinine levels and abnormal glomerular filtration rate (GFR). A patient is said to have end-stage kidney disease (ESKD) when their estimated glomerular filtration rate (eGFR) ranges drops from over 90 ml/min/1.73m^2^ to less than 15ml/min. It is said to progress at a slower rate over years [5].

The progression of diabetic nephropathy shall be managed by determining the amount of protein excreted in the urine (proteinuria) and the amount of creatinine in the blood serum (serum creatinine) [6]. High blood glucose level affects circulation of blood and metabolism in the body, resulting in the excess production of reactive oxygen species (ROS) (chemically highly reactive molecules containing oxygen). These reactive oxygen species damage the glomerular network of kidneys leading to the release of albumin in the urine (called albuminuria) [7, 8].

The pathophysiology of the glomerulus in diabetic nephropathic kidneys shall be studied from three types of cells: endothelial cells, the podocytes and the mesangial cells. In the glomerulus, all of these cells remain in physical contact with one another and communicate chemically at a distance. As a result, all three types of cells in DN will be aberrant [9].

As the disease progresses, the damage to the glomerular filtration barrier (GFB) in the glomeruli keeps increasing. The fenestrated endothelium, the glomerular basement membrane, and the epithelial podocyte make up this barrier, which is extremely selective to filtration of blood that enters the glomeruli. The GFB permits only smaller molecules, water, and extremely small proteins to pass through (albumin does not pass through the intact GFB due to its higher molecular size) [10]. Proteinuria is caused by damage to the glomerular membrane, which permits bigger proteins in the blood to seep into the urine. Kimmelstiel–Wilson nodules are periodic-acid Schiff positive nodules that form when abnormally large volumes of mesangial matrix are deposited. The generation of cytokines and advanced glycation end products as a result of high blood sugar has also been suggested as a possible mechanism for the development of diabetic nephropathy [11].

An chemical molecule known as anethole is commonly used as a flavouring ingredient. It’s a phenylpropene derivative found in essential oils. Phenylpropene is a sort of aromatic chemical found in abundance in nature. Several studies in recent years have revealed multiple beneficial effects of anethole for human health [12].

According to a study, the Western diet is becoming increasingly processed junk food and fat, with a well-established link between excessive consumption of this sort of food and recent rises in obesity and type 2 diabetes prevalence. Both type 1 and type 2 diabetes produces alterations in glucose transport in the kidney, but junk food or a high-fat diet generates changes that are very similar to those seen in type 2 diabetes [13]. Accordingly the ameliorative impact of an anethole-rich fraction from aromatic herbs was studied in this study by inducing diabetic nephropathy in rats by feeding junk food.

## Materials and methods

### Collection of Plant materials and drug solutions

The aromatic herbs such as Foeniculum vulgare (fennel), Pimpinella anisum (anise), Mentha piperita (mentha) and Anethumgraveolens (dill) were collected and were authenticated by the Director of PARC (Plant Anatomy Research centre), Medicinal Plant Research Unit, West Tambaram, Chennai, India and a specimen was deposited in PARC.

The standard drug Metformin (Brand name: Metlad SR 500 mg tablet 10’s; Company: Glaxosmithkline) was used. The isolated compound from the collected herbs was used as the test compound. The other chemicals and reagents utilized in this study were analytical grade and purchased from a commercial source.

### Preparation of Metformin drug solution: [14]

Metformin (Brand name: Metlad SR 500 mg tablet 10’s; Company: GlaxoSmithKline) (0.33 g/kg) was administered to the rats. About 33 mg of Metformin was dissolved in 1000ml of distilled water and was administered to rats through I.P. route.

### Extraction and quantification of anethole rich fraction: [15]

Anethole enriched herbs such as fennel, anise, mentha and dill were selected. 500 g of each herb were taken and shade dried to preserve the volatile constituents. Ground herbs were put into a Soxhlet apparatus attached to a solvent flask after being transferred to a filter paper extraction thimble. The proportion of plant material to ethanol solvent was 1:10 (w/w). The extractions were carried out at intervals ranging from 10 to 120 min.

HPLC analysis was carried out at SRM Institute of Science and Technology’s Interdisciplinary Institute of Indian System of Medicine (IIISM) utilizing an HPLC system with a UV-VIS detector and a thermostatted flow cell, and at two wavelengths of 190 to 370 nm and 371 to 600 nm, respectively. An integrator was used to record the detector signal. As a column, the C18 block heating-type Shim-pack VP–ODS (4.6 mm inside diameter, 150 mm length) was employed, with a particle size of 5 μm. For the separation of anethole, methanol: water (75:25 v/v) was employed as a solvent, with a flow rate of 1.0 ml/min and a column temperature of 30 °C. The injection volume was 40 l, and detection was done at a wavelength of 346 nm. The preliminary phytochemical screening of the extracted anethole rich fraction was performed.

### Junk food preparation

Blended chips (250 g), chocolate chips cookies (260 g), potato chips (80 g), peanut butter (260 g), choco powder (260 g), Rodents lab diet (400 g), cheese, chili flakes (3 g), refined flour (10 g), Ajinomotto (monosodium glutamate) (2 g), food color (0.5 g) were taken and are made as a fine powder. From this 5 g of this mix was taken and dissolved in 50ml of distilled water and 1ml of this was then administered to the animals using an oral gavage twice a day for a period of 8 weeks.

### Experimental animals and experimental procedure [16]

Wistar rats, male, SPF, weighing 200–220 g, provided by Tamil Nadu Veterinary and Animal Sciences University (TANUVAS), Madhavaram milk colony, Chennai, Tamilnadu, India. The Animal Ethical Committee (AEC) approved this study, with the approval number being IAEC/213/2019.

24 male wistar rats about 2–3 months old were selected and 6 rats were grouped as control and were fed with normal rat feed for a period of 8 weeks. Other rats were fed with junk food. Induction period was for 8 weeks. After 8 weeks, blood samples from all rats were collected and blood glucose levels were checked. If BG levels ranges from 250 to 300 mg/dl with observable symptoms of polydypsia, polyphagia and polyurea, the rats were considered as diabetic nephropathy induced rats.

The 18 DN induced rats were randomly divided into 3 groups with 6 animals in each group, one as standard and the other two for the administration of low dose (50 mg/kg) and high dose (100 mg/kg) of the isolated drug fraction twice a day for a period of 30 days.

On 10th, 20th and 30th days of treatment, the BG of the rats was measured. On the 30th day, rats were individually placed in metabolic cages for 24 h, urine samples were collected for the measurement of urine output, creatinine and urine protein. Blood samples were collected for the measurement of fasting blood glucose, serum creatinine and blood urea nitrogen (BUN) from retro-orbital plexus on the same day.

Rats were sacrificed at the end of the treatment period to excise the kidneys and pancreas. The excised kidneys and pancreas were kept in 10% formalin and later processed in paraffin for consequent histochemical studies. Remaining kidney tissues were homogenized and the tissues were stored for measurement of levels of Xanthine Oxidase (XO), Xanthine Dehydrogenase (XDH) and Super Oxide Dismutase (SOD), Catalase and Glutathione at -80 °C.

### Measurement of renal, biochemical parameters [17, 18]

On the 10th, 20th, and 30th days post treatment, rats’ fasting blood glucose levels were assessed. Urine samples from rats were taken on the 30th day of sample administration by placing the rats individually in metabolic cages for a period of 24 h for the analysis of urine protein content and creatinine. 2 h after the last sample was administered, blood samples were drawn and centrifuged to detect the serum levels of GSP, TG, TC, IA, HDL, LDL, insulin, MDA, GSH, SOD, BUN, and creatinine.

### Histopathology [19, 20]

The weight, renal index, and histological parameters were calculated by, the animals’ kidneys and pancreas which were extracted and fixed in a 10% neutral buffered formalin solution, dried in ethanol, and fixed in paraffin. A rotary microtome was used to cut sections of around 5 mm thickness, which were subsequently stained with hematoxylin and eosin (H & E) dye for histological analysis.

Renal index = kidney weight/rat weight.

### Statistical analysis [21]

Statistical analysis was carried out using Graphpad Prism 6 and one-way analysis of variance (ANOVA). The results were considered significant if p < 0.05, and statistical values were expressed as mean SEM.

## Results

### Phytochemical screening

The preliminary screening of phytochemical components of the extracted anethole rich fraction showed the presence of following phytochemical compounds. (Table 1)

**Table 1:** Phytochemical screening

S. NO.	COMPONENTS	OBSERVATION
1	Alkaloids	Positive
2	Steroids	Positive
3	Sterols	Positive
4	Tannins	Positive
5	Flavonoids	Positive

### Identification of anethole by HPLC

**Fig. 1:**
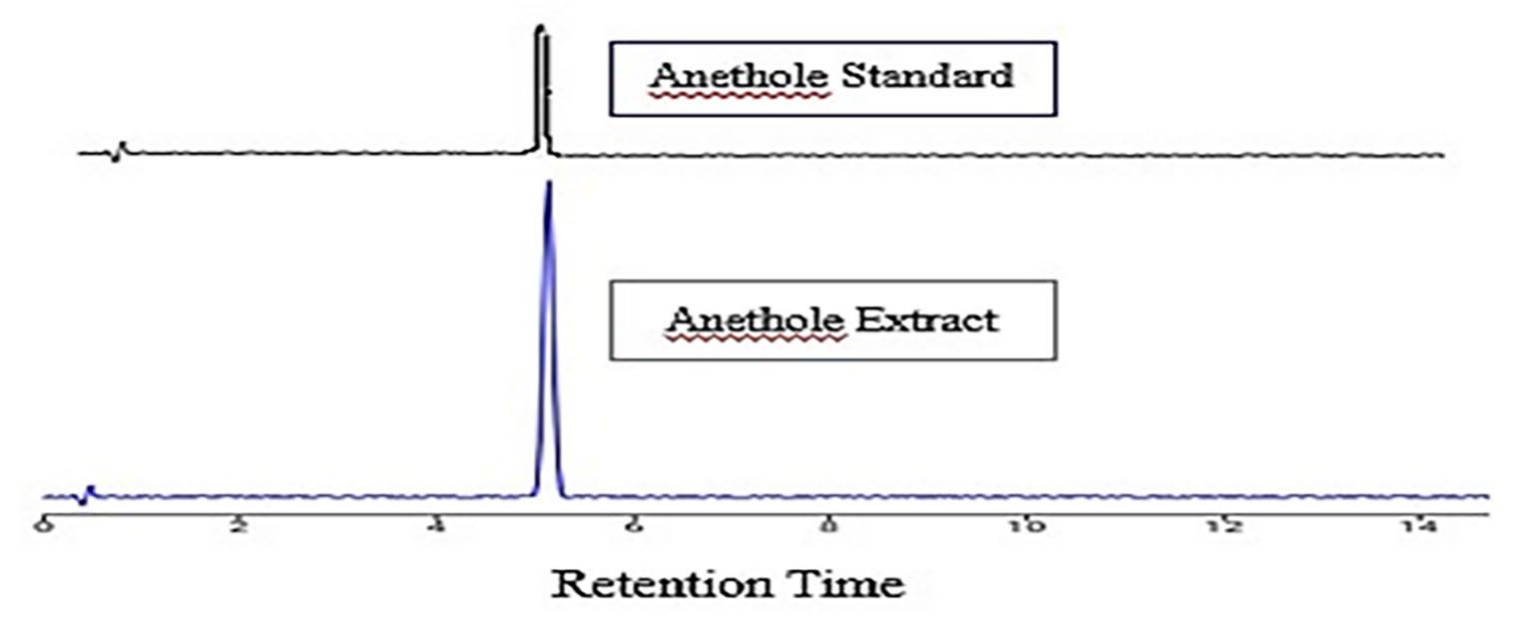
HPLC chromatogram of anethole

The extracted anethole was identified by comparing it with the standard anethole using High Performance Liquid Chromatography. The peak’s retention time was observed to be similar in both the standard and test compounds. (Fig. 1)

### Effect of anethole rich fraction on the body weight

The body weight was found to be decreased in the diabetic nephropathy induced animals during the treatment. The least weight loss was observed in the control group. There was an increase in the body weight of animals treated with the extract. (Table 2)

**Table 2:** Body weight changes

GROUPS	INITIAL BODY WEIGHT*	FINAL BODY WEIGHT*
Control	242 ± 2.12	297 ± 2.55
Junk food fed group	263 ± 2.58	233 ± 2.81
DN induced + low dose of anethole rich fraction	282 ± 1.67*	250 ± 3.42*
DN induced + low dose of anethole rich fraction	276 ± 2.32*	259 ± 3.65*

Results are depicted as mean ± SEM. The value comparison was made between the control and Group II, III, IV (*p < 0.001)

### Effect of anethole rich fraction on the blood glucose level

The blood glucose level in the junk food fed group was increased when compared to the other groups. Upon administration with anethole rich fraction, the animals showed a decrease in the blood glucose levels. The treatment groups had a control on the blood glucose level. (Table 3)

**Table 3:** Blood glucose levels

GROUPS	0 WEEK*	2 WEEKS*	4 WEEKS*	8 WEEKS*
Control	113 ± 1.57	121 ± 1.32	118 ± 2.18	120 ± 1.53
Junk food fed group	118 ± 10.9	411 ± 10.6	562 ± 11.3	584 ± 12.21
DN induced + low dose of anethole rich fraction	119 ± 11.2*	345 ± 12.6*	317 ± 10.9*	229 ± 10.81*
DN induced + low dose of anethole rich fraction	-	397 ± 11.9*	332 ± 10.4*	214 ± 10.12*

Results of each group (n = 6) represents are depicted as mean ± SEM. The value comparison was made between the control and Group II, III, IV (*p < 0.001)

### Effect of anethole rich fraction on insulin level

In comparison to normal control rats (15.13 ± 0.13µ IU/ml), junk food fed diabetic nephropathy caused rats had a significant decrease in fasting insulin levels (7.04 ± 0.21µ IU/ml). Anethole-rich fraction administration for 30 days resulted in a considerable increase in serum insulin levels. (Table 4)

**Table 4:** Serum insulin level

GROUP	SERUM INSULIN LEVEL*
Normal	15.13 ± 0.13µ IU/ml
Junk food fed	7.04 ± 0.21µ IU/ml
Anethole rich fraction(50 mg/kg)	11.21 ± 0.18µ IU/ml*
Anethole rich fraction(100 mg/kg)	15.09 ± 0.15µ IU/ml*

Results of each group (n = 6) represents are depicted as mean ± SEM. The value comparison was made between the control and Group II, III, IV (*p < 0.001)

### Effect of anethole rich fraction on the levels of GSP and lipid profile

**Fig. 2:**
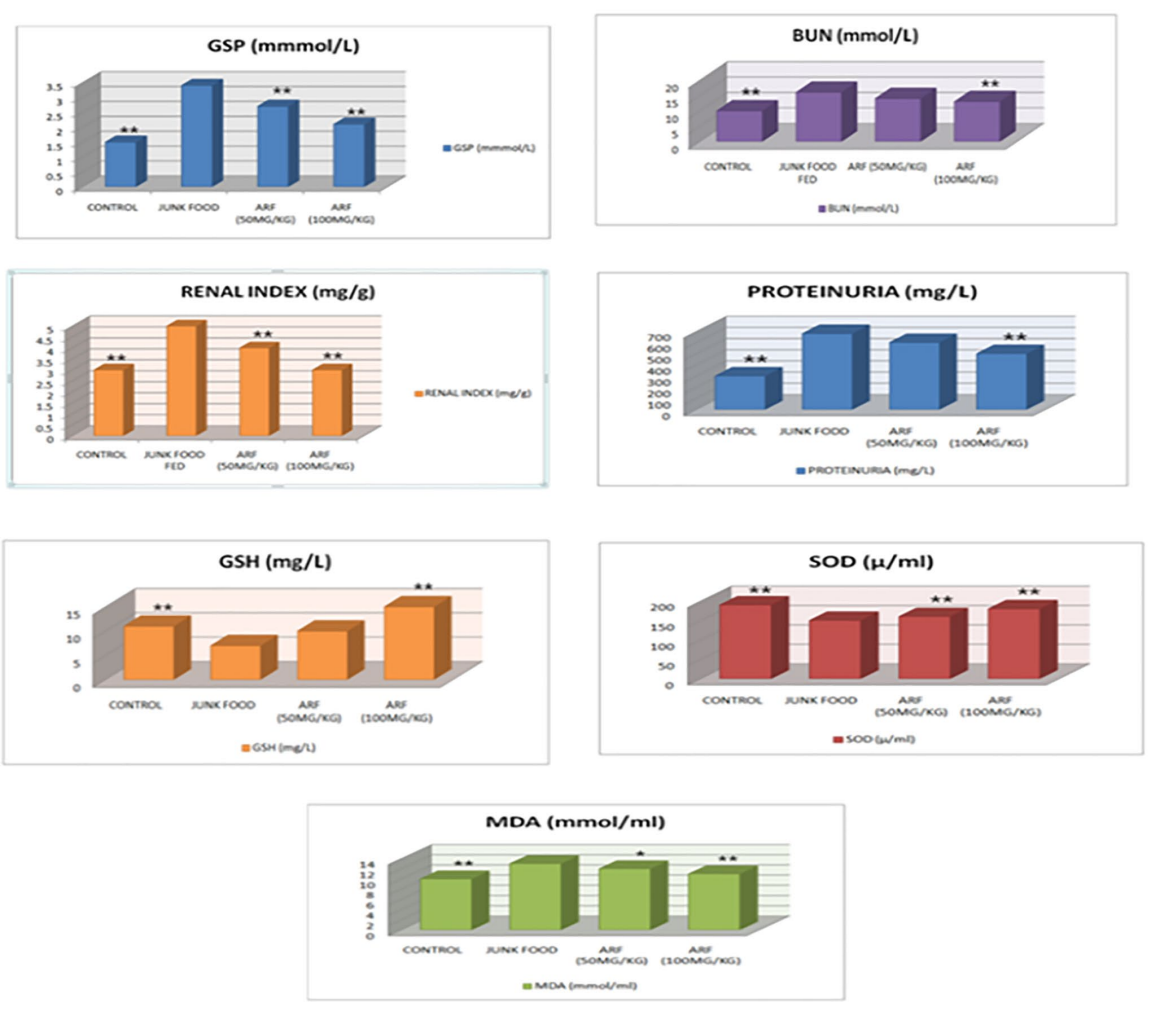
Effect of ARF on GSP, RI, BUN, PU, GSH, SOD, MDA all group rats (mean ± SD value, n=6

On comparing with the model group, negative control group, high dose and low dose groups, it be could found that the test drug treated groups could considerably decrease the levels of GSP. (Fig. 2)

Total cholesterol (TC), low density lipoprotein (LDL), very low density lipoproteins (VLDL), and triglyceride (TG) levels in diabetic nephropathic rats were dramatically raised after 30 days of treatment with anethole rich fraction. With injection of anethole rich fraction 50 and 100 mg/kg, PG levels were lowered by 24.72, 39.36, 48.00, and 55.05%, respectively. In DN rats, the levels of VLDL and LDL were likewise dramatically lowered after receiving 50 and 100 mg/kg of anethole rich fraction, respectively. In addition, high density lipoproteins (HDL) were shown to be severely reduced in DN rats, and treatment with anethole rich fraction 50 and 100 mg/kg dramatically raised HDL levels. (Table 5)

**Table 5:** Effect of anethole rich fraction on lipid profile

PARAMETERS	TC (mg/dl)	TG (mg/dl)	LDL (mg/dl)	VLDL (mg/dl)	HDL (mg/dl)
Control	102.5 ± 2.64	70.34 ± 2.14	14.14 ± 2.05	32.95 ± 0.654	54.64 ± 0.45
Junk food fed group	285.4 ± 2.45*	205.27 ± 3.17*	173.67 ± 2.25*	91.18 ± 0.15**	25.06 ± 0.239**
Anethole rich fraction- low dose (50 mg/kg)	131.42 ± 4.08**	91.89 ± 1.25*	48.97 ± 3.21**	41.18 ± 0.364***	40.04 ± 0.19***
Anethole rich fraction- low dose (100 mg/kg)	114.24 ± 2.24***	62.78 ± 1.01**	35.89 ± 2.46***	34.25 ± 0.82*	44.05 ± 0.604***

Results of each group (n = 6) represents are depicted as mean ± SEM. The value comparison was made between the control and Group II, III, IV (*p < 0.001, **p < 0.01, ***p < 0.05)

### Effect of anethole rich fraction on renal function

Over the course of the trial, junk food given DN rats showed a significant increase in urea (97.96 mg/dl), uric acid (16.04 mg/dl), and creatinine (4.11 mg/dl) levels. The effect of treatment with low and high dosages of anethole rich fraction was similar to that of the control group (Table 6). In addition, low and high doses of anethole rich fraction significantly reduced the renal index of DN rats (P < 0.01) (Fig. 2), as well as the blood urea nitrogen (BUN) (Fig. 2) and proteinuria levels of DN rats. (Fig. 2).

**Table 6:** Effect of anethole rich fraction on renal function

PARAMETERS	UREA (mg/dl)	URIC ACID (mg/dl)	CREATININE (mg/dl)
Control	32.82 ± 0.296	5.72 ± 0.148	0.83 ± 0.002
Junk food fed group	96.69 ± 0.185*	15.04 ± 0.126*	4.72 ± 0.046*
Anethole rich fraction- low dose (50 mg/kg)	51.84 ± 0.145***	7.62 ± 0.016*	1.71 ± 0.132**
Anethole rich fraction- low dose (100 mg/kg)	42.27 ± 0.597***	6.63 ± 0.117**	1.29 ± 0.085**

Results of each group (n = 6) represents are depicted as mean ± SEM. The value comparison was made between the control and Group II, III, IV (*p < 0.001, **p < 0.01, ***p < 0.05)

### Effect of anethole rich fraction on anti-oxidant enzymes and lipid peroxidation

It can be observed that the levels of superoxide dismutase (SOD) and reduced glutathione were observed to be abnormally high in junk food fed diabetic nephropathic rats when compared to normal control rats (Fig. 2). Continuous administration with anethole rich fraction significantly reduced the levels.

### Histopathological evaluation

#### Effect of anethole rich fraction on pancreatic tissue

**Fig. 3:**
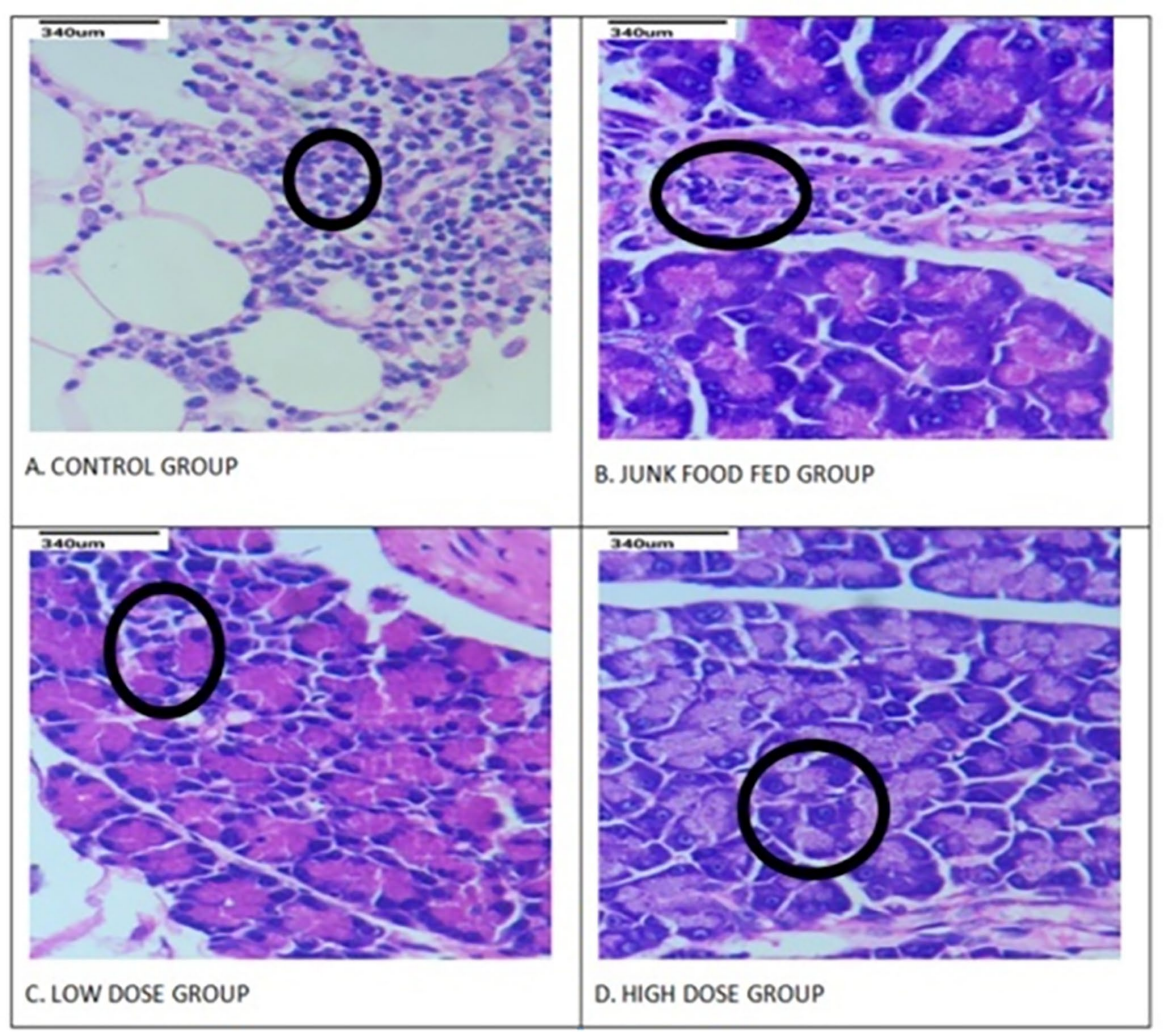
Effect of anethole rich fraction on pancreatic tissue of DN rats

There were few pathological changes in the tissues of pancreas (p < 0.01) identified on comparing with the control and DN induced groups (Fig. 3). The islet nuclei of junk food-fed rats were found to be thick, accompanied with cytoplasm atrophy in the majority of islet cells. In DN-induced rats, the high and low dose groups demonstrated a substantial reduction in morphological alterations of pancreas tissues when compared to the negative control group (p < 0.01).

#### Effect of anethole rich fraction on kidney tissue

**Fig. 4:**
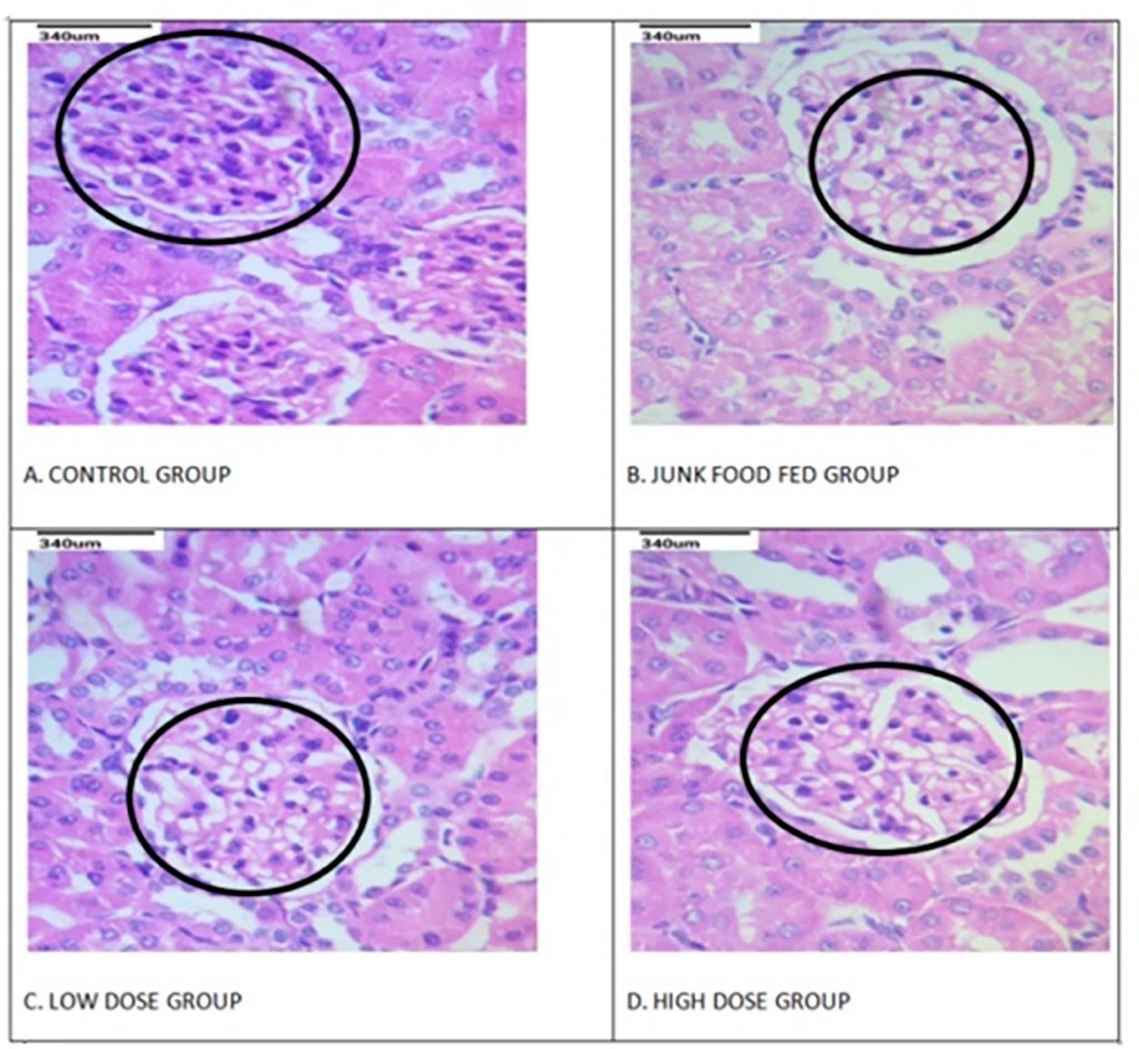
Effect of anethole rich fraction on kidney tissue of DN rats

It is observed that there are significant pathological changes in the tissues of kidney of junk food fed group (Fig. 4). The glomerular cells of the negative control group were found to be highly proliferated with the disappearance of renal cyst cavities. Meanwhile it is found that the high and low dose groups have brought down the pathological changes of the kidney tissue at a significant range (p < 0.01). The expansion of glomerular cells was not found to be very noticeable in the low dose group, despite the minor constriction of the renal cyst, whereas the high dose group was able to clearly diminish the degenerative changes in kidney tissue (p < 0.05).

## Discussion

This study has demonstrated that the anethole rich fraction modified the progression of disease in diabetic nephropathy rats by improving the renal functions, oxidative stress levels, glycometabolismand morphological alterations of kidney and pancreas. This study was also conducted to learn the influence of lifetime exposure to a junk food diet in Wistar male rats. Concretely, the protective effect of anethole- rich fraction was found beneficial in the treatment of diabetic nephropathy in rats.

Previous studies which used the same junk food diet recipe found that the diet induced weight gain in animals when compared to those with normal feed [22, 23]. The earliest morphological anomalies in diabetic nephropathy are the area glomerular basement membrane thickening, accumulation of the extracellular matrix and expansion of the mesangium. Albuminuria is now being investigated as a discrete entire mark in the metabolic syndrome. [24, 25].

Glycometabolism, aberrant production of different cytokines, renal damage, which includes free radical action and oxidative stress are all contributors in the etiology of diabetic nephropathy. Diabetic Nephropathy is due to hemodynamic and structural changes including glomerular hyper filtration and hypertrophy, thickening of glomerular basement membrane, mesangial membrane expansion, tubulointestitial fibrosis, glomerular hypertrophy, podocyte and renal cell death, creatinine and protein excretion, renal dysfunction, irregular glomerular filtration rate, action of Angiotensin II by the over expression of AT1R receptors and imbalance in sex steroid hormone levels [26]. The assessment of blood glucose level variations is the major index of evaluation model and efficacy since glycometabolism disorder is one of the most important pathological manifestations of diabetes. Insulin resistance is a defining feature of type 2 diabetes, and insulin resistance or sensitivity loss can prevent glucose from being converted to fat, resulting in brain dysfunction in a variety of ways [27]. Insulin is still one of the most commonly prescribed medications for diabetes mellitus. Advanced research is critical for discovering novel compounds with great therapeutic value and fewer adverse effects.

In this study, diabetic nephropathy was produced in the experimental mice by feeding them junk food. Junk food is rich in fat, saturated fat, energy density, fructose, and glycemic index, but low in fibre, vitamins A and C, and calcium, according to nutritional research. Junk food is found to have highly refined carbohydrates and less in fibre which is the main cause for inflation in glycemic index. High glycemic index food triggers post- prandial elevation leading to a proportionate secretion of insulin by β cells. Feed with a high glycemic index accelerates fat deposition in rats, resulting in obesity. Glycemic index is linked not only to obesity but also to type II diabetes mellitus. Through glucotoxicity, lipotoxicity, and over stimulation, a high-glycemic index diet may impair cell function. [27]. As a result of this cell aberration, insulin release is reduced, resulting in hyperglycaemia and prolonged hyperglycemias was considered to be the pre condition for the advancement of diabetic complications. [28]

To build a base for producing the aforesaid test drug, the efficacy of anethole- rich fractions from diverse aromatic herbs was confirmed from five aspects: glucose metabolism and insulin resistance, oxidative stress, lipid metabolism, renal function, and pathological alterations of excised organs. This study looked at the impact of junk food consumption on albuminuria, fibrosis, renal oxidative stress, podocyte loss, and insulin resistance. Increasing renal cellular oxidative stress appears to be caused by increased adiposity. Early podocyte loss was identified in histological investigation, with loss of podocyte cells being the first event, followed by albuminuria [29].

According to studies, junk food consumption causes renal oxidative stress, fibrosis, albuminuria, podocyte loss, and insulin resistance. Increased levels of cellular oxidative stress indicators are linked to these pathological alterations in the kidney. The development of microalbuminuria (urinary albumin excretion rate of 20–200 mg/min) is the first clinical symptom of DN. If left untreated, roughly 20–40% of T2DM patients would develop overt albuminuria, and about 20% will acquire end stage renal disease (ESRD) within the first twenty years of disease onset [30]. The introduction of junk food to rats resulted in an increase in blood glucose levels in this study. Catabolic reactions accelerate as a result of hyperglycemia, resulting in muscle loss and, as a result, a large reduction in animal body weight. In diabetic rats, therapy with anethole- rich fraction improved body weight.

Hyperglycemia is always preceded by anomalies in lipoprotein metabolism resulting in the increase in the levels of TG, TC, VLDL, LDL and reduction in the levels of HDL which evidence the high movement of fats from adipose tissue due to the utilization of glucose from peripheral tissues [31]. When anethole-rich fraction was given to diabetic rats, the levels of TG, TC, VLDL, and LDL were found to decrease dramatically, while the level of HDL was found to increase, as compared to diabetic control rats.

In diabetic rats, the levels of uric acid, urea, and creatinine in the blood are indicators of DN development.In this study, the biochemical values were found to be in excess in diabetes rats as compared to normal rats. Upon treatment with the extracted compound, the values of these biochemical variables were found to be significant when compared with normal rats, thus showing a protective role against DN or an adjournment in its progression.

Hyperglycaemia and oxidative stress are two interconnected variables that contribute to the onset of diabetes.The antioxidant enzymes (GSH and SOD) were reported to be protective against the advancement of diabetes [32]. When diabetic rats were compared to non-diabetic rats, the levels of these enzymes were shown to be lower. The rise in thiobarbituric acid-reactive substances (TBARS), a lipid peroxidation indicator in diabetic rats, could be related to an increase in oxygen free radical levels. Because of its antioxidant action, delivery of the chemical resulted in a significant drop in TBARS levels and an increase in SOD and GSH levels in the kidney in this investigation.

Increase in the free radical level has been linked with non-enzymatic glycation of proteins, liquid peroxidation and oxidation of glucose which contributes towards diabetes mellitus leading to nephropathy [33]. The advanced glycation end products produced by these free radicals cause mesangial cell damage, resulting in mesangial enlargement and basement membrane thickening. As a result, inhibiting these free radicals and preventing the production of advanced glycation end products could be a possible therapeutic strategy for DN treatment [34]. The current investigation shows that anethole-rich fraction has a possible inhibitory effect on the production of free radicals and the advanced glycation end product.

The generation of free radicals and advanced glycation end products surge in diabetic rats’ kidneys, resulting in the multiplication of glomeruli with mesangio capillary and advancement of hemodynamic parameters, according to histological observations [35]. Administration of anethole-rich fraction in rats with diabetic nephropathy showed a healing effect in the structure and anatomy of their kidney. The antioxidant activity of anethole may play a role in improving damaged kidney cells and reducing pathological alterations, which may be attributed to the phenolic and flavonoid components found in anethole.

Evidence suggests that anethole is a natural bioactive molecule having anti-inflammatory, anticancer, chemopreventive, neuroprotective, spasmolytic, hypotensive, antithrombotic, immunomodulatory, and antidiabetic properties in humans. It may be a safe therapy option for a variety of chronic conditions, including skin and lung inflammatory disorders, cancer, type 2 diabetes and neurological problems. The regulation of various signalling pathways, particularly NF-kB, TNF-a, and MAPK pathways, appears to represent the underlying mechanisms for anethole effectiveness [36].

## Limitations

Susceptibility to diabetic nephropathy with albuminuria and the development of glomerular and tubulointerstitial lesions varies depending on the genetic background and strain of each mouse model. As a result, the validation of an animal model that reproduces human diabetic nephropathy will greatly aid our understanding of the genetic pathways that lead to diabetic nephropathy development. Hence, in addition to traditional diabetic rat models, more refined mouse models that target additional genes for deletion, either alone or in combination, may be valuable for diabetic nephropathy research [37].

## Conclusions

It can be concluded that anethole has the ability to vitiate diabetic nephropathy in junk food induced diabetic rats. The high dose of anethole (100 mg/kg) could reach the intervention affect by improving glycometabolism, lipid metabolism, oxidative stress level, insulin resistance, renal function, restoration of antioxidant enzymes, inhibition of generation of free radicals and the advanced glycation end product and improving the morphological alterations of pancreas and kidneys. The outcomes of this experiment again confirmed for the treatment of DN and the effect was found to be better in the high dose group (100 mg/kg), aided the research foundation for the subsequent development and clinical use of an anethole rich fraction. However, more biochemical and histological research is needed to determine the underlying mechanism of action of anethole in diabetic nephropathy rats.

## Abbreviations

ARF: Anethole Rich Fraction
BUN: Blood Urea Nitrogen
DN: Diabetic Nephropathy
GSH: Glutathione Reductase
GSP: Glycated Serum Protein
H&E: Hematoxylin& Eosin
MDA: Malondialdehyde
SOD: Superoxide Dismutase
